# Reading and Visual Search: A Developmental Study in Normal Children

**DOI:** 10.1371/journal.pone.0070261

**Published:** 2013-07-19

**Authors:** Magali Seassau, Maria-Pia Bucci

**Affiliations:** 1 e(ye)BRAIN, Ivry-sur-Seine, France; 2 UMR 676 Inserm - Paris 7, Hôpital Robert Debré, Paris, France; University of Leicester, United Kingdom

## Abstract

Studies dealing with developmental aspects of binocular eye movement behaviour during reading are scarce. In this study we have explored binocular strategies during reading and during visual search tasks in a large population of normal young readers. Binocular eye movements were recorded using an infrared video-oculography system in sixty-nine children (aged 6 to 15) and in a group of 10 adults (aged 24 to 39). The main findings are (*i*) in both tasks the number of progressive saccades (to the right) and regressive saccades (to the left) decreases with age; (*ii*) the amplitude of progressive saccades increases with age in the reading task only; (*iii*) in both tasks, the duration of fixations as well as the total duration of the task decreases with age; (*iv*) in both tasks, the amplitude of disconjugacy recorded during and after the saccades decreases with age; (*v*) children are significantly more accurate in reading than in visual search after 10 years of age. Data reported here confirms and expands previous studies on children's reading. The new finding is that younger children show poorer coordination than adults, both while reading and while performing a visual search task. Both reading skills and binocular saccades coordination improve with age and children reach a similar level to adults after the age of 10. This finding is most likely related to the fact that learning mechanisms responsible for saccade yoking develop during childhood until adolescence.

## Introduction

A good eye movement control, in particular saccades and fixations, is essential for reading. Several studies have examined ocular motor behaviour in children and adults subjects. Buswell [1], Rayner [2] and McConkie et al. [3] showed that younger children have longer and more frequent fixations, smaller saccades, and frequent regressive saccades (leftward saccades). In line with these findings, a review article, Levy-Schoen and O′Regan [4] reported developmental aspects of reading and they described that reading speed increased with increasing of children's age and reading skill capabilities. Moreover, the duration of fixations decreased also with age reaching adult level at about 11 years old. Reading capabilities also increase as children grow, leading to an improvement of these ocular motor performance [5]. Another important difference bewteen children and adults is the so-called ‘perceptual span’ area, i.e. the section of text from which a subject can extract useful information from reading. Rayner [2] reported that this area is smaller in beginner readers than in proficient adult readers. This difference is consistent within a child population: 7 year-olds process a smaller area of text during fixation than older children (11 year-olds). This result could partially explain why younger children are slower readers. Interestingly, a reduced perceptual span has also been suggested to be the cause of reading difficulties in dyslexics [6].

Until 2006 the majority of research dealing with eye movements in reading was limited to measuring movements from only one eye. However, reading is an activity requiring saccades and vergence eye movements: horizontal saccades bring the eyes to successive words but the vergence angle between the two eyes needs to be adjusted to the distance of the word for appropriate fusion of the two retinal images to take place. Bucci & Kapoula [7] found that the binocular coordination of saccades while reading single words in 7 year-old children is significantly worse than in adults; such poor coordination is observed both when reading single words and when fixating a LED light. The authors suggested that fine binocular motor control, as is needed for reading, develops via learning mechanisms based on the interaction between the saccadic and the vergence systems. Poor binocular control in young readers could explain the long fixation durations observed and would interfere with the process of learning to read. Developmental aspects of binocular saccade coordination have been previously reported by Fioravanti, Inchingolo, Pensiero & Spanio [8]. These developmental aspects in children were noted by recording horizontal saccades to LED-targets. The authors compared saccade characteristics of 3 groups of subjects: young children aged 5–9, older children aged 11–13 and adults. The authors showed similar ocular motor behaviour between the older children and the adult group, i.e., smaller saccade disconjugacy. In contrast, the younger group of children reported larger saccade disconjugacy. The authors attributed this effect to a "poor compensation of mechanical asymmetries of the orbital planes existing in young children".

Bassou, Granié, Pugh, & Morucci [9] were the first to record binocular eye movements in 10 year olds while reading a text. The authors showed that saccades of the two eyes can be highly disconjugate in children of this age, suggesting that Hering's law [10] where both eyes are well yoked because they receive equal innervation, is not always obeyed during reading. Recall that Hering postulated that the brain sends a unique command to each eye, so that they move as a uniform organ. Bassou and collaborators pointed out that poor binocular control in children could interfere with learning to read. Note however, that due to the low resolution of the recording system used (i.e. an EOG device), these results were only on percentage of asymmetry of the amplitude of saccades between the two eyes.

Cornelissen, Munro, Fowler & Stein [11] compared eye movements during reading word lists in a group of twenty children aged 9–10 and ten young adults. Eye movements were recorded by an infra-red system (IRIS, SKALAR). They found that children showed significant poorer binocular coordination when fixating words than the adults.

Blythe et al. [12] compared binocular coordination in twelve children (aged 7 to 11) and in twelve young adults (aged 18–21). They measured the binocular coordination at the beginning and at the end of the fixation, and found that children showed significantly larger disconjugate fixations than adults. Furthermore, fixations in children were more divergent than in adults who showed more frequent convergent fixations. Taken together, these differences between children and adults are in line with the hypothesis that children's ocular motor control is immature.

A recent study from our group (Bucci, Nassibi, Gerard, Bui-Quoc, & Seassau [13]) explored the quality of binocular coordination during reading and during visual search in groups of dyslexic and non dyslexic children of various ages. For non dyslexic children, we reported that the disconjugacy measured during and after the saccade was significantly smaller in 10–12 year-olds than in 8–9 year-olds. Furthermore, young children made smaller saccade amplitudes, and tended to fixate more often and for longer than in older children. Such ocular motor behaviour has been observed both while reading and in a visual search task, suggesting an immaturity of the ocular motor saccade and interaction of vergence systems.

Based on these studies, the quality of binocular coordination during and after saccades seems to be under-developed in children. It should be noted however that the mentioned studies examined a small number of children or examined children with a large range of ages. The purpose of the present study is to further examine binocular measures of saccades during reading and during visual search tasks in a large population of normal readers aged from 6 to 15 years, and compare these results with those from a group of adults. Our driving hypothesis, in line with Cornelissen et al. [11] and Blythe et al. [12] and with our previous works on reading [13]; [7], is that binocular performance during reading will improve with age. The collected data could also prove useful as a reference for any further studies examining ocular motor development in children with reading difficulties.

## Materials and Methods

### Subjects

Sixty-nine children (aged 6 to 15) and 10 adults (aged 24 to 39) participated in the study. For an easier presentation of their clinical and visual characteristics, participants were divided into five groups of children depending on their age and scholastic level: 15 children aged 6–7 years (mean age: 7.0±0.1, first grade of French primary school); 15 children aged 8–9 years (mean age: 8.5±0.1, second and third grade of French primary school); 16 children aged 10–11 years (mean age: 10.8±0.1, fifth and sixth grades of French school); 11 children aged 12–13 years (mean age: 13.0 ± 0.1, seventh and eighth grade of French secondary school); 12 children aged 14–15 years (mean age: 14.5±0.1, ninth grade of French secondary school), and one group of adults. An ANOVA performed on the mean age showed that the groups were significantly different from each other (F_(5,73)_ = 262.84, p<0.0001). Participants had to satisfy the following criteria to be included in the study: no known neurological or psychiatric history, no history of reading difficulty, no visual impairment or difficulty with near vision. Children underwent both the similarity test of the WISC IV (assessing verbal capability by abstracting criteria common to two objects and by excluding differences) and the matrix test of the WISC IV (assessing logic capability). All children tested had normal verbal (10.4±0.4) and logic (11.9±0.5) capabilities (normal range is 10 ± 3, as reported in Wechsler intelligence scale for children fourth edition, 2004).

Participants underwent both a sensorial and motor ophthalmologic examination (mean values showed in Table 1). All participants had normal binocular vision (mean value of 51 s of arc), which was evaluated with the TNO random dot test. Visual acuity was normal (≥20/20) for all participants. The near point of convergence was normal for all participants (mean value of 2 cm). Heterophoria at near distance (i.e. latent deviation of one eye when the other eye is covered, using the cover-uncover test) was normal for all children tested (≤ exophoria of 3.5 prism D). Moreover, an evaluation of vergence fusion capability using prisms was done at near distance. The divergence and convergence amplitudes were also normal for all participants.

**Table 1:** Clinical characteristics of the six groups of participants examined.

	TNO	NPC	Heterophoria	Divergence	Convergence
**6–7 years**	54	2	2	14	34
**8–9 years**	45	2	2	14	35
**10–11 years**	53	2	3	14	38
**12–13 years**	51	3	2	13	34
**14–15 years**	50	2	2	16	35
**Adults**	54	3	2	15	34

Mean values for: Stereoacuity test: TNO, measured in seconds of arc; Near point of convergence: NPC, measured in cm; Heterophoria at near distance, measured in prism diopters; Vergence fusional amplitudes (divergence and convergence) at near distance, measured in prism diopters.

The investigation adhered to the principles of the Declaration of Helsinki and was approved by the Institutional Human Experimentation Committee (CPP Ile de France I, Hôpital Hotel-Dieu). Written consent was obtained from the children's parents after an explanation of the experimental procedure.

### Ocular motor paradigms

Stimuli were presented on a 22 inches PC screen, with a resolution of 1920×1080 and a refresh rate of 60 Hz. Although it is well known that intermittent illumination could affect saccade accuracy and visual assessment [14], this refresh rate was sufficient to assure a normal saccade performance.

The reading and visual search tasks are similar to those used by Bucci et al. [13] and are described below.

#### Reading

**Figure 1:**
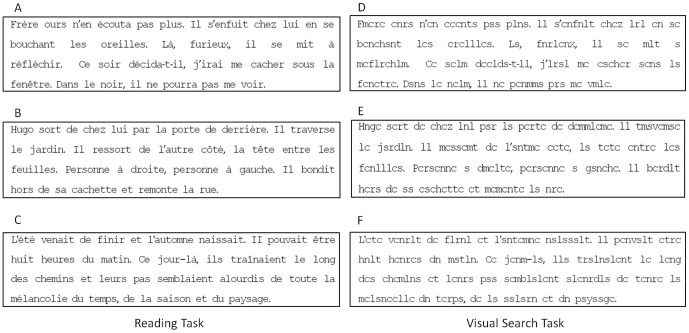
Stimuli used for the reading (A, B and C) and visual search (D, E and F) tasks for each group of children children: age 6–9; 10–12, and 13 and more respectively.

Subjects were asked to read a text of four lines from a children's book. The paragraph contained 40 words and 174 characters. The text was 29° wide and 6.4° high; mean character width was 0.5° and the text was written in black “courier” font on a white background. Each age group had to read a different text. Figure 1 show each of these texts: an extract from “*Jojo Lapin fait des farces*”, Gnid Bulton, Hachette Ed., for 6 to 9 year-olds (Fig. 1A); an extract from “*Bagarres à l’école*”, Marc Cantin et Eric Gasté, Castro Cadet ed., for 10 to 12 year-olds (Fig. 1B); and an extract from “La guerre des boutons”, Louis Pergaud. Folio Ed., for 13 year old children and adults (Fig. 1C). Participants were asked to read the text silently. When they were finished, they raised a finger and were asked to describe the text. This allowed the researchers to check that the text had been read and understood. The texts used were from three different books that are frequently used by French teachers in different class levels (7–9, 10–12, and over 13 years old). As said in our previous work [15] we chose these age-specific texts to ensure that all words were well-known and easily understood by the children. Note that after the task was completed, the researcher asked the child a few questions in order to verify that he/she read the text and understood it.

#### Visual search

This task used the same texts as in the reading task, with one crucial difference: all vowels were replaced by consonants (see Figure 1D, 1E, 1F for the texts for 6 to 9 year-olds, 10 to 12 year-olds and 13 to15 year-olds, respectively). Children were asked to count silently the number of ‘r’s occurring in the text. When they were done, they raised a finger and were asked to report this number to the researcher. Table 2 shows the percentage of the number of ‘r’s counted in the text by each group of participants tested, and the corresponding post-hoc group comparisons.

**Table 2:** Percentage of the number of ‘r’s counted in the visual search task, by the six groups of participants tested, and post-hoc comparisons by groups.

	Percentage of ‘r’ (SE)	Post-hoc group comparisons
**6–7 years**	76.7 (3.8)	p = 0.46
**8–9 years**	80.7 (3.8)	
		p = 0.06
**10–11 years**	91.3 (3.7)	
		p = 0.57
**12–13 years**	94.5 (4.5)	
		p = 0.84
**14–15 years**	95.8 (4.3)	
		p = 0.85
**Adults**	97.0 (4.7)	

In both tasks, stimuli were presented without time limitations. The recording of each task stopped when the child raised one finger to indicate that they were finished reading/counting.

### Eye movement recordings

Eye movements were recorded with the Mobile Eyebrain Tracker (Mobile EBT**®**, e(ye)BRAIN, www.eye-brain.com), an eye-tracking device CE marked for medical purposes. The Mobile EBT® benefits from a high frequency camera that allows it to record both the horizontal and vertical eye positions independently and simultaneously for each eye. Recording frequency was set up to 300 Hz. The precision of this system is typically 0.5°.In a controlled setting as in the one we used, it reaches 0.25° (see www.eye-brain.com). The recording system does not obstruct the visual field and the calibrated zone covers a horizontal visual angle of ± 22° (see Lions et al., 2013).

### Procedure

Children were seated in a chair in a dark room and used a headrest to avoid any head movement. Viewing was binocular with a viewing distance of 60 cm. Calibration was done at the beginning of each eye movement recording, for each eye during binocular viewing. The best calibration could be an haploscopic arrangement [16]. However, it should be noted that binocular vision was normal for all children tested (see stereoacuity scores in Table 1), suggesting that they were fixating targets with both eyes. A previous study from [17] comparing normal and strabismic children confirmed that in the absence of strabismus either type of calibration (under monocular or binocular viewing) was valid.

During the calibration procedure, children were asked to fixate a grid of 13 points (diameter 0.5 deg) mapping the screen. Point positions in horizontal/vertical plans were: −20.9°/12.2°; 0°/12.2°; 20.9°/12.2°; −10.8°/6.2°; 10.8°/6.2°; −20.9°/0°; 0°/0°; 20.9°/0°; −10.8°/−6.2°; 10.8/−6.2°; −20.9°/−12.2°; 0°/−12.2°; 20.9°/−12.2°. Each calibration point required a fixation of 250 ms to be validated. A polynomial function with five parameters was used to fit the calibration data and to determine the visual angles. After the calibration procedure, the reading or visual search tasks were presented to the child. Duration of each task was kept short (lasting a couple of seconds) to avoid any head movements and to ensure an accurate measurement of eye movements (see for details [15]).

### Data analysis

An ANOVA was performed with the six age groups as inter-subject factor and clinical orthoptic values as within subject factors.

For ocular motor data, calibration factors for each eye were determined from the eye positions during the calibration procedure. The software MeyeAnalysis (provided with the eye tracker, e(ye)BRAIN, www.eye-brain.com, France) was used to extract saccadic eye movements from the data. It automatically determines the onset and the end of each saccade by using a built-in saccade detection algorithm. The algorithm used to detect saccades is adapted from [18]. All saccades with an amplitude superior to 1 degree were detected. All detected saccades were checked by the researcher and corrected/discarded if necessary.

The number and the amplitude of progressive saccades (prosaccades, from left to right) and regressive saccades (backward saccades, from right to left) and the duration of fixations between each saccade were analyzed. The time to perform each task was also analyzed and was determined by the delay between the first and the last saccade. In both tasks (reading and visual search), binocular coordination was defined for each saccade and each fixation was recorded. We examined the amplitude of the disconjugate components during each saccade (left eye - right eye). The disconjugacy was measured as the change in vergence between the beginning and the end of each saccade. We also examined the disconjugate component of each post-saccadic fixation period (see [13]).

Data were analyzed using different multiple linear regression models using the number of saccades, the amplitude of saccades (in degrees), the duration of fixations (in ms) and the duration of task (in seconds). Given that saccade disconjugacy depends on the saccade amplitude, the values of disconjugacy during and after the saccades were presented as the ratio of the disconjugacy on the saccade amplitude (in percentage). The predictor variable for each test was the participant's age (in year and months). Linear regressions were performed for children only, and presented on corresponding graphs as dotted lines. We also measured the correlation coefficient between the saccadic disconjugacy and the post-saccadic fixation disconjugacy.

Finally, ANOVAs were performed with the six age groups as inter-subject factor and the type of task (reading vs visual search) as within subject factor. We considered the effect of a factor to be significant when the p-value was below 0.05.

## Results

### Eye movement pattern during reading and visual search

#### Number of saccades

**Figure 2:**
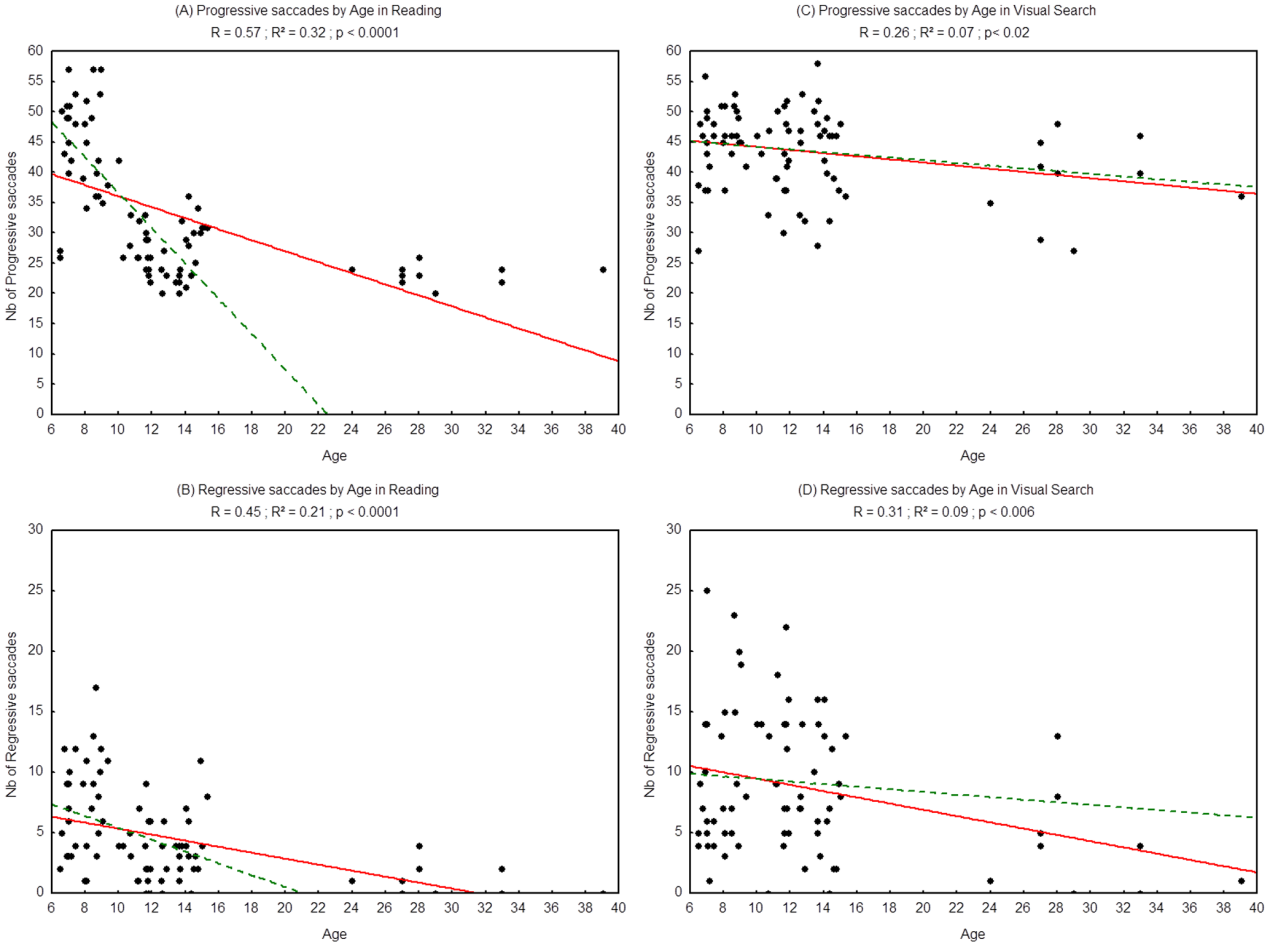
Number of progressive saccades during reading (A), visual search (C) and number of regressive saccades during reading (B) and visual search (D). Lines represent the corresponding regressions. Dotted lines represent the regressions for children (from 6 to 15 years old) only, 2A: R^2^ = 0.53, p<0.0001; 2B: R^2^ = 0.13, p<0.003; 2C: R^2^ = 0.009, p = 0.45; 2D: R^2^ = 0.003; p = 0.67.

Figure 2 shows the number of progressive and regressive saccades assessed during reading (2A & 2B) and visual search (2C & 2D) as a function of age for each participant examined, and the regression line observed in each case. There was a significant effect of age in the reading task: the number of saccades decreased as age increased (R^2^ = 0.32, p<0.0001 and R^2^ = 0.21, p<0.001, respectively for the progressive and regressive saccades). There was also a significant effect of age on the visual search task as the number of progressive saccades (R^2^ = 0.07, p<0.02) and the number of regressive saccades (R^2^ = 0.09, p<0.006) decreased with age.

#### Amplitude of saccades

**Figure 3:**
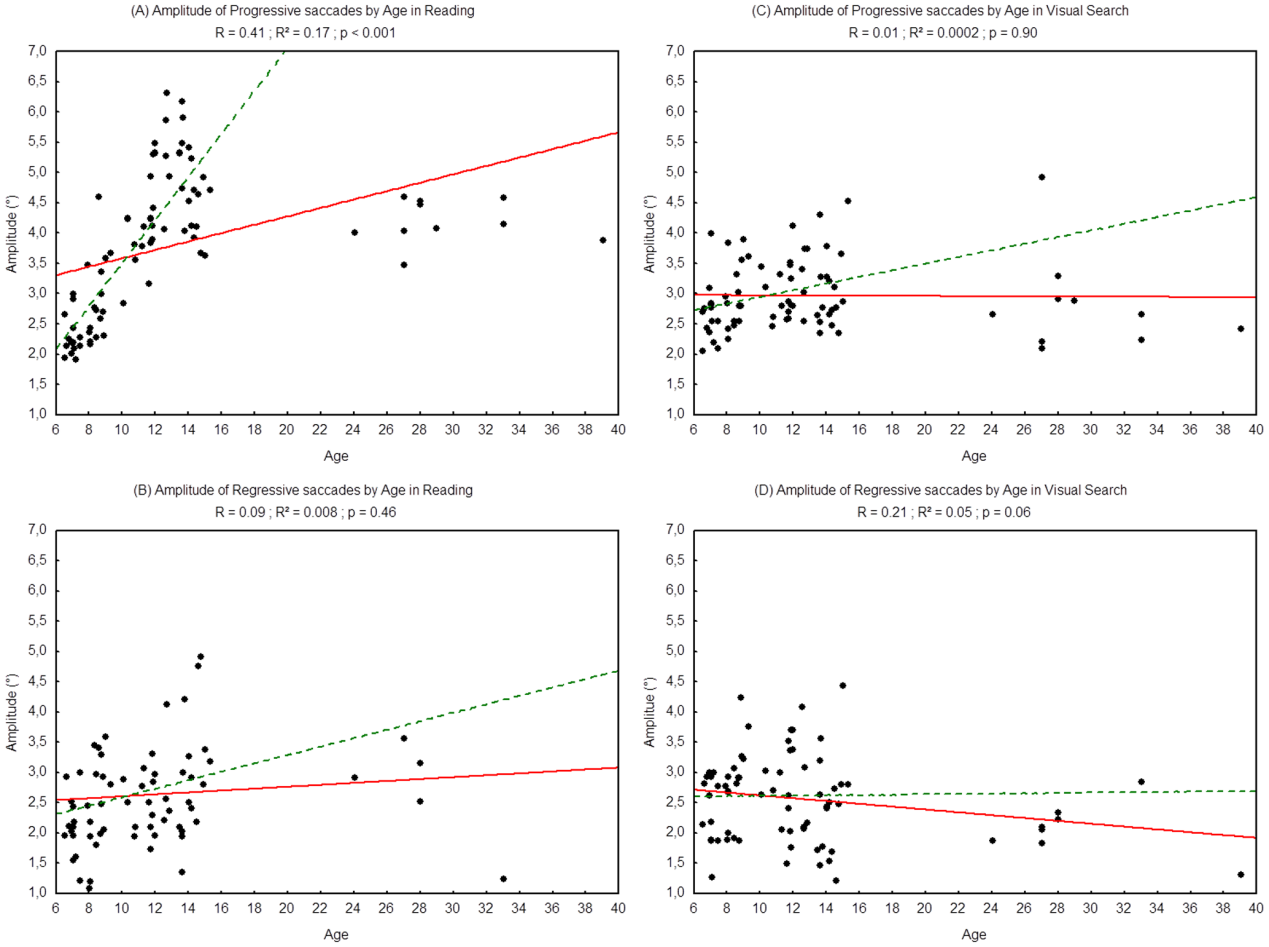
Amplitude of progressive saccades during reading (A), visual search (C) and amplitude of regressive saccades during reading (B) and visual search (D). Lines represent the corresponding regressions. Dotted lines represent the regressions for children only (from 6 to 15 years old), 2A: R^2^ = 0.65, p<0.0001; 2B: R^2^ = 0.04, p = 0.10; 2C: R^2^ = 0.08, p<0.02; 2D: R^2^ = 0.0001; p = 0.94.

Figure 3 shows the mean amplitude of saccades (progressive and regressive) assessed during reading and visual search tasks for each participants. There was a significant effect of age: the amplitude of progressive saccades increased with age in the reading task (R^2^ = 0.17, p<0.001) but not in the visual search task (R^2^ = 0.0002, p = 0.90). We found no effect of age on the amplitude of regressive saccades, neither in the reading task (R^2^ = 0.008, p = 0.46) nor in the visual search task (R^2^ = 0.05, p = 0.06).

#### Duration of fixations

**Figure 4:**
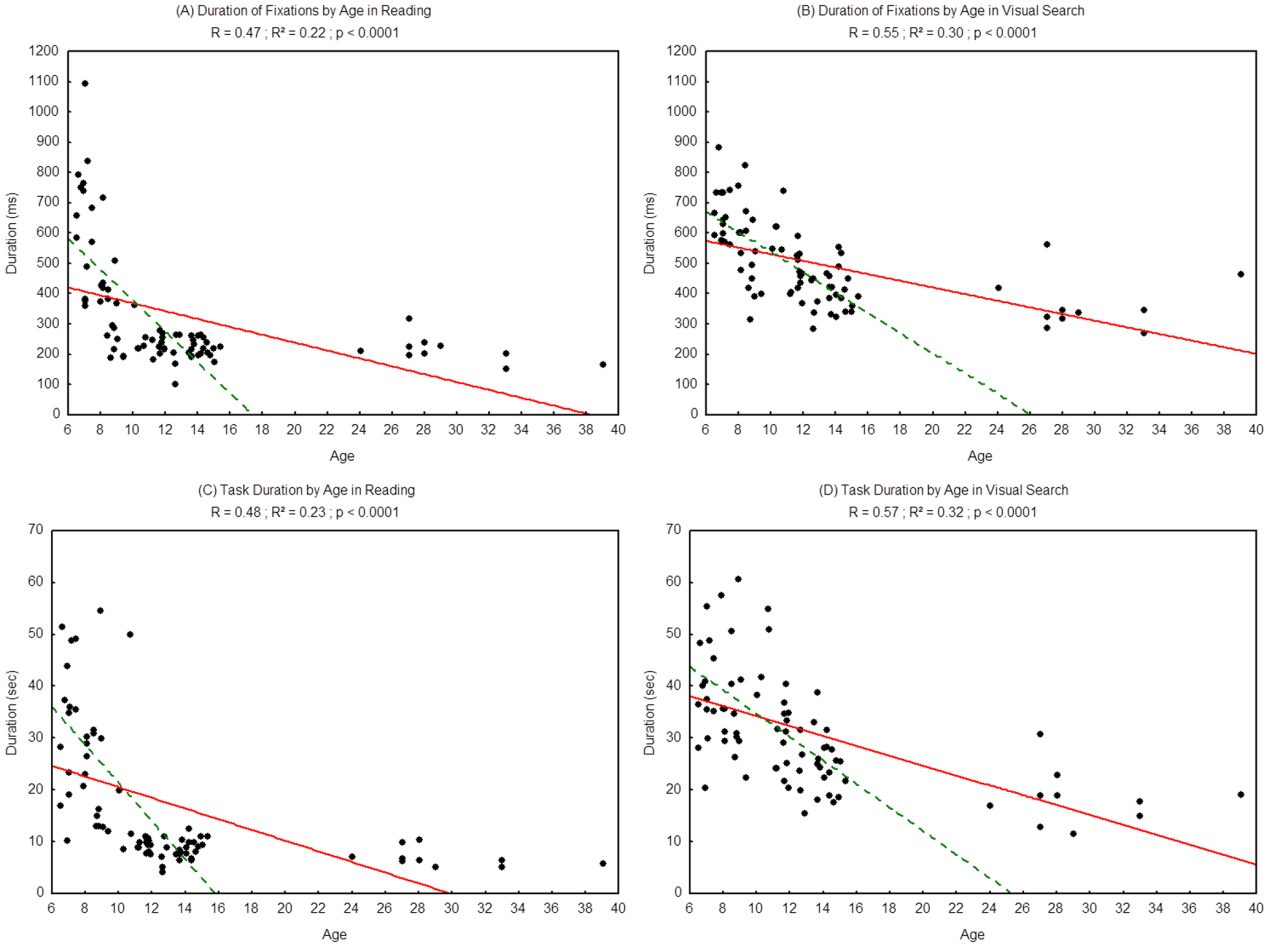
Duration of fixations during reading (A) and during visual search (B) and task duration in reading (C) and visual search (D). Lines represent the corresponding regressions. Dotted lines represent the regressions for children only (from 6 to 15 years old), 2A: R^2^ = 0.51, p<0.0001; 2B: R^2^ = 0.48, p<0.0001; 2C: R^2^ = 0.45, p<0.0001; 2D: R^2^ = 0.45; p<0.001.

In order to better understand the participants fixation behaviour, we also measured the average duration of fixations, which is the time period between two saccades (Figure 4A for reading and 4B for visual search). We found a significant effect of age on the duration of fixations, which decreased with age in both tasks (reading: R^2^ = 0.22, p<0.0001; visual search: R^2^ = 0.30, p<0.0001).

#### Total task duration

We measured the period between the first saccade and the last fixation period, or total task duration. Figure 4C and 4D show this duration assessed for every participant in the reading task and visual search task respectively. We found a significant effect of age in both the reading task (R^2^ = 0.23, p<0.0001) and the visual search task (R^2^ = 0.32, p<0.0001). In both cases, the mean task duration decreased as age increased.

### Binocular Coordination during reading and visual search

#### Disconjugacy during the saccades

**Figure 5:**
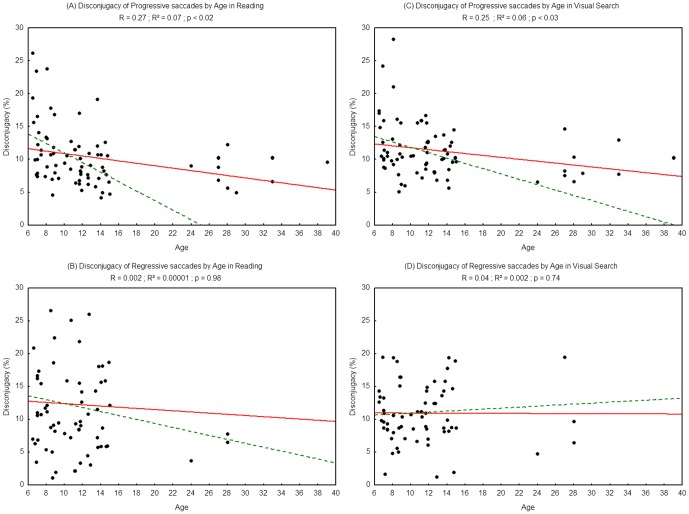
Saccadic disconjugacy of progressive saccades in the reading (A) and visual search tasks (C) and disconjugacy during the regressive saccades in the reading (B) and visual search tasks (D). Lines represent the corresponding regressions. Dotted lines represent the regressions for children only (from 6 to 15 years old), 2A: R^2^ = 0.11, p<0.006; 2B: R^2^ = 0.006, p = 0.53; 2C: R^2^ = 0.07, p<0.03; 2D: R^2^ = 0.007; p = 0.50.

Figure 5 shows the disconjugacy observed during saccades. For both tasks we found a significant effect of age on disconjugacy in progressive saccades (R^2^ = 0.07, p<0.02 and R^2^ = 0.06 p<0.03 respectively for reading and visual search task) but not in regressive saccades (R^2^ = 0.0001, p = 0.98 and R^2^ = 0.002, p = 0.74 respectively for reading and visual search): the disconjugacy of progressive saccades decreased with age.

#### Disconjugacy of post-saccadic fixation period

**Figure 6:**
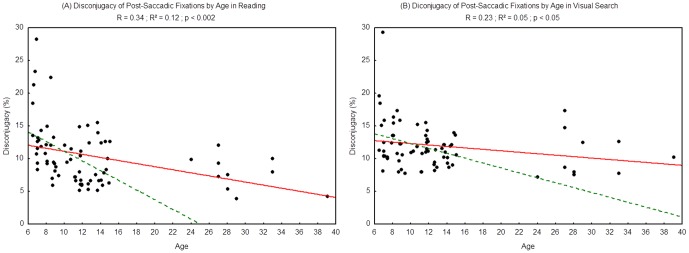
Mean value of post-saccadic fixation disconjugacy during reading (A) and visual search (B). Lines represent the corresponding regressions. Dotted lines represent the regressions for children only (from 6 to 15 years old), 2A: R^2^ = 0.13, p<0.003; 2B: R^2^ = 0.09, p<0.02.

The values of disconjugacy measured during the post-saccadic fixation period are shown in Figure 6. For both tasks we found a significant effect of age (R^2^ = 0.12, p<0.002 and R^2^ = 0.05, p<0.047 respectively for reading and visual search): the disconjugacy of post-saccadic fixation period decreased with age.

#### Sign of disconjugacy

**Figure 7:**
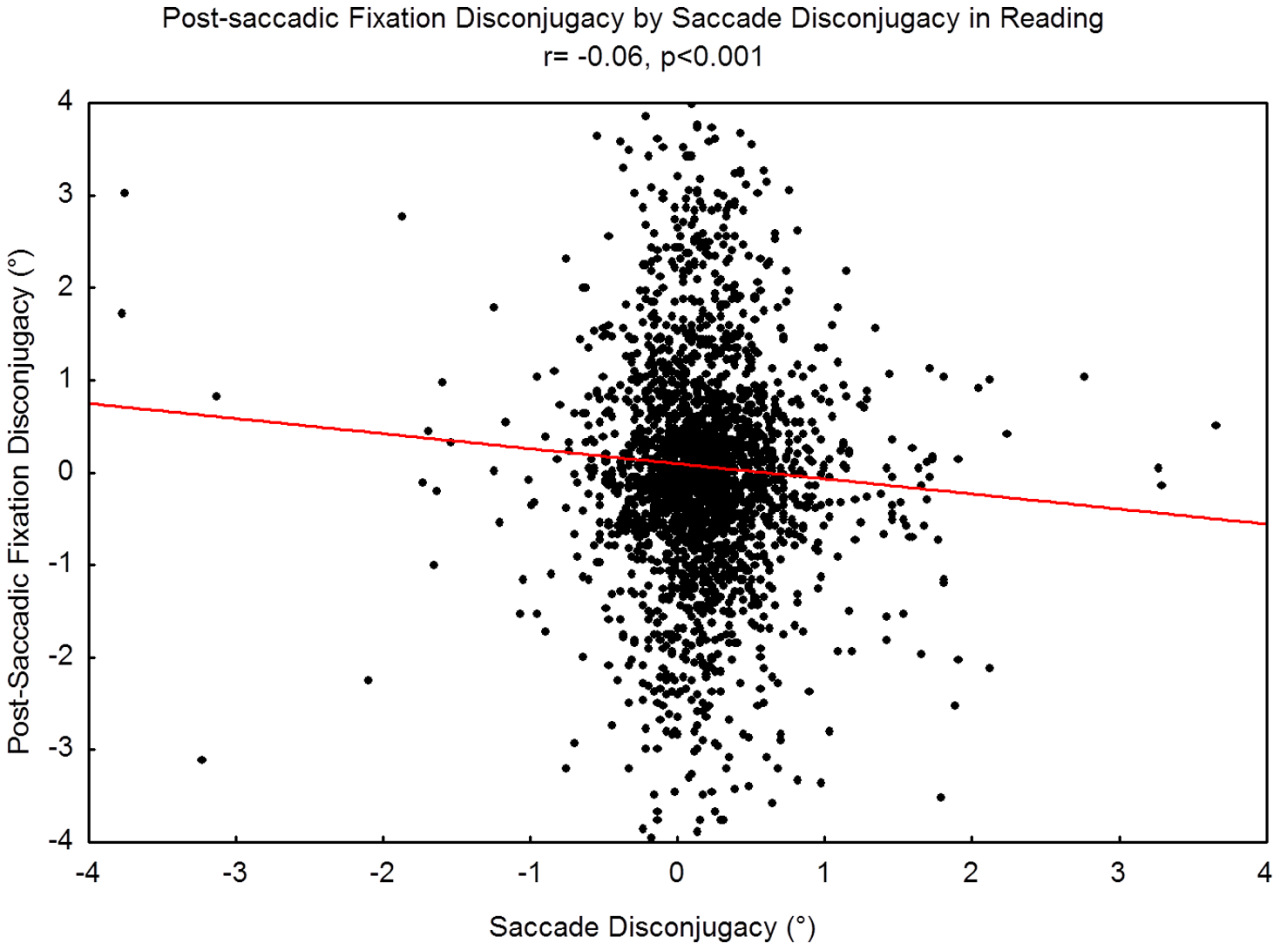
Correlations between saccadic disconjugacy and post-saccadic fixation disconjugacy for each saccade analyzed.

In Figure 7, the saccadic disconjugacy is plotted versus the post-saccadic fixation disconjugacy for each saccade of each participant examined in the reading task. We found a significantly negative Pearson's correlation in reading (r  = −0.06; p<0.001), indicating that the disconjugacy of the saccades, which is divergent for the majority of the cases, is followed by convergent disconjugacy during the post-saccadic fixation period, thus reducing binocular disparity.

### Type of Task: Reading versus visual search

We focused on the development of automatic processing in reading by comparing the reading task and the visual search task. Children were plotted by age and school levels: 6–7 years (first grade); 8–9 years (second and third grade); 10–11 years (fifth and sixth grade); 12–13 years (seventh and eighth grade); 14–15 years (ninth grade); and compared to a group of normal adults (aged between 24 and 39).

#### Number of progressive and regressive saccades (Figure 8A and 8B)

**Figure 8:**
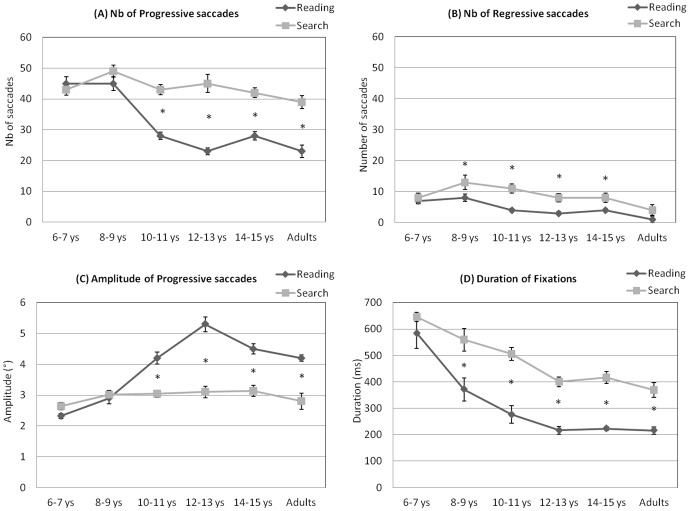
Comparisons of reading and visual search task for the six groups of participants tested on: number of progressive saccades (A), number of regressive saccades (B), amplitude of progressive saccades (C) and duration of fixations (D). Vertical lines indicate the standard error. Asterisks indicate that value is significantly different between reading and visual search task.

We found a significant task effect, with more saccades observed in the visual search task than in the reading task (F_(1,72)_ = 130.57, p<0.0001) and a significant interaction between task and group (F_(4,72)_ = 10.89, p<0.0001). Post hoc comparisons showed no difference between the tasks in the 6–7 year-old and 8–9 year-old groups. For all other groups of children and adults, the number of progressive saccades was smaller in reading than in the visual search task (all ps<0.0001). The number of regressive saccades was smaller in the reading task than in the visual search task for children aged between 10 and 15 years (all ps<0.002) but there was no difference in adults (p = 0.08).

#### Amplitude of progressive saccades (Figure 8C)

Given that regressive saccades were not age-dependent (see figure 3), we only focused on the amplitude of progressive saccades. We found a significant task effect, (F_(1,72)_ = 121.84, p<0.0001), with smaller saccade amplitudes in the visual search task than in the reading task and a significant interaction between task and group (F_(5,72)_ = 22.11, p<0.0001). Post hoc comparisons showed no difference between both tasks in the 6–7 year-old and 8–9 year-old groups. For all groups of older children and adults, the amplitude of progressive saccades was larger in reading compared to the visual search task (all ps<0.001).

#### Duration of fixations (Figure 8D)

There was a significant effect of task on the fixation durations (F_(1,73)_ = 116.54, p<0.0001), with longer durations of fixations in the visual search task than in the reading task. We also found a significant interaction between task and group (F_(5,73)_ = 4.93, p<0.001). Post hoc comparisons showed no difference between reading and visual search for the 6–7 year-old group (p = 0.47) and fixations were shorter in reading than in visual search (all ps<0.001) for the four other groups of children and adults.

#### Task duration

We found a significant task effect (F_(1,72)_ = 167.95, p<0.001) with the duration of fixations as the visual search took longer to complete than reading. The interaction between task and group was also significant (F_(5,72)_ = 6.56, p<0.001). Post hoc comparisons showed no difference between reading and visual search for the 6–7 year-old group (p = 0.17), while task duration in reading was shorter compared to visual search (all ps<0.001) for the four other groups of children and adults,

In summary, comparisons between the two tasks showed that between 6 and 9 years of age, the reading task is performed in a very similar fashion to the visual search task. After 10–11 years of age, children are significantly more accurate and faster in reading than in visual search, as with adults.

## Discussion

The aim of the study was to further explore the binocular coordination of saccades while reading a text and and while performing a visual search task in a large population of children and compare these results to those obtained from a group of normal adults. The most important findings are discussed below.

### Saccade and fixation duration characteristics during reading

Our findings concerning binocular data on the number of saccades and fixation duration during reading are in line with findings previously reported on ocular motor behaviour from McConkie et al. [3] and more recently from Blythe et al. [12] showing that children's reading skills develop with age. The present study on a large population of children showed that during reading young children have smaller saccades, frequent regressive saccades and that their fixations are longer. This ocular motor behaviour is typically observed in children learning to read and for whom reading skills are still immature. With age, children's reading capabilities improve and they learn to read by making larger progressive saccades, fewer regressive saccades and shorter fixations; reading a text for older children becomes a simple and quite rapid task similar to adults.

The improvement of reading skills could be due to cortical development. Luna, Velanova, & Geier [19] reported that the activity of some cortical areas involved in saccadic eye movements (e.g. frontal and parietal cortex) is lower in young children than in adults and increases until adolescence. Furthermore, temporal and parietal structures are involved in linguistic processes and they develop during childhood [20]; [21]. A recent fMRI study from Olulade et al. [22] reported differences between children and adults during word processing in the anterior left occipito-temporal cortex, providing evidence of developmental course of those regions. Our findings in reading are in line with this developmental hypothesis. Indeed, both the number of saccades (progressive and regressive), the duration of fixations, and the duration of the task decrease with age independently of the task, suggesting an improvement in the performance of saccades. Note, however, that brain imaging studies during reading in a large population of children will need to explore further such issue.

### Disconjugacy during and after saccades in reading and visual search task

This study shows that disconjugacy measured during and immediately after the saccade (during the post-saccadic fixation period) decreases with age. This finding is in line with previous studies from our group [7] in normal 7 year-olds and more recently from [13] comparing binocular saccade coordination in normal and dyslexic children during reading. Fioravanti, Inchingolo, Pensiero & Spanio [8] were the first to show that saccades were highly discoordinated in young children and that such disconjugacy decreased with age reaching adult values at about 11–12 years. Fioravanti's study [8] was run on a small number of subjects (6 children) and measured horizontal saccades to target-LEDs at a relatively far viewing distance (1 m). Despite different experimental conditions, our results are in line with Fiovaranti et al's [8], suggesting that central ocular motor learning mechanisms responsible for saccade yoking are still immature in young children and that they develop with training and visual experience. The binocular coordination of saccades in older children (>10 years old) becomes similar to those reported in adult subjects. Our data is also in line with Blythe et al. [12] who compared the binocular coordination of saccades during a reading task in twelve children (7 to 11 years) and twelve young adults (18 to 21 years).

Based on these studies we can make some hypothesis on how and where good quality binocular coordination is reached in humans. According to Lewis et al. [23], we can hypthosize that the fine control of binocular saccade coordination is based on an efficient relationship between the motor command of the saccades and the vergence subsystems at the premotor level. This hypothesis is based on studies from our group [13]; [15]; [24] of different types of child populations showing poor vergence fusional capabilities (i.e., dyslexic children, children with strabismus and children with vergence insufficiency). Indeed, recall that during the reading task (an activity done at near distance) a correct convergence command strictly linked with the saccade command is needed in order to adjust the visual axes of the two eyes at the distance of the word for appropriate fusion of the two retinal images. All child populations with poor vergence capabilities (as those previously cited) showed poor binocular saccade control. This hypothesis, however, needs further exploration by testing the quality of binocular saccade coordination before and after orthoptic vergence training.

Another important aspect of binocular saccade coordination is the disconjugacy before and after the saccades. Blythe et al. [12] reported that fixations in children were more frequently divergent while adults showed more frequent convergent fixations during the post-saccadic period. It is well known that in order to decrease or eliminate the divergent disconjugacy during the saccades, adults make convergent post-saccadic fixations (see [25]). Our data on the correlation between the saccade disconjugacy and the post-saccadic fixation disconjugacy shows that in most cases the divergent saccade disconjugacy is reduced afterwards by convergent post-saccadic fixations. This is the normal pattern due to the abducting-adducting eye asymmetry reported by Collewjin et al. [25] in adult subjects. The origin of such a stereotypical pattern in adults is still not clear and Collewjin and collaborators did not exclude a central or peripheral origin for this mechanism. Collewijn, Erkelens, & Steinman [26] suggested that the divergent saccade disconjugacy could be a useful strategy in the natural environment to respond quickly with a divergence during an eye movement. Our data, in contrast with Blythe et al. [12], shows that this ability is already present in 6 year-olds. It is most likely that this mechanism does not need adaptation to visual experience to work correctly and, for this reason it could have more peripheral than central/cortical origin.

### Reading versus visual search task

The reading and the visual search tasks had different demands on visuo-perceptual, attentional and spatial processing. Consequently one could expect to observe different ocular motor behaviour in these two tasks. This was the case except for the disconjugacy measured after the saccades. The data on the number, amplitude of saccades and duration of fixations showed significant differences between the different groups of children and adults. In general we can say that younger children (6–7 and 8–9 year old groups) display similar ocular motor behaviours in both tasks while older children similar to adults do not. We suggest that when reading capabilities are not yet developed children perform both tasks in a similar way. In contrast, when children have better developed reading skills, they accomplish both tasks differently, which is reflected in their ocular motor behaviour. In older children, the number and the duration of the fixations are different in both tasks because the underlying cognitive processes are different. Indeed, in a visual search task, children are asked to identify and count a single target (r), and have to inspect each letter closely, whereas in the reading task they can easily skip letters without disrupting their reading (skipping letters being part of a well-developed reading ability). For these reasons, for older children as with adults, reading becomes an easier and faster task to perform than a visual search.

Finally, we have to point out that we did not observe any difference between both tasks in terms of the quality of binocular coordination of saccades. This data confirms and expands our previous work in relation to normal as well as dyslexic children (Bucci et al. [13]) showing that reading a text does not interfere with the quality of binocular coordination. However, the present study is in contrast with that of Heller and Radach [27]. Heller and Radach [27] reported that adult subjects showed a large disconjgacy during the post-saccadic fixation period when they read a text. The authors advanced the hypothesis that the material of the reading task influences the binocular coordination of saccades. They concluded that binocular motor control while reading normal text is poor, most likely because the semantic process is easier with normal text and can be achieved even in the absence of perfect binocular motor control. In line with this hypothesis Kirkby, Blythe, Drieghe, & Liversedge [28] reported poor binocular coordination in dyslexic children during reading but not during a non-linguistic scanning task, suggesting that reading processing difficulties associated to reduced engaged attention, could affect binocular coordination in dyslexia.

## Conclusion and Futures Studies

In summary, the data reported here suggests an immaturity of the binocular coordination of saccades during reading as well as during visual search tasks in the youngest children. At 6 or 7 years of age, an age at which children start to learn to read, not only are reading skills immature (as previous studies had shown, see review of Rayner [5]) but saccades from the two eyes are also unyoked. We report this disconjugacy during and after the saccades both in reading and in a visual search task. In other words, we do not observe a change in the properties of binocular coordination depending on the type of task. Further studies exploring reading and other visual tasks in which linguistic processes do not occur (for example scanning of simple dot stimuli as used by Kirkby, Blythe, Benson, & Liversedge [29]) in large child populations will be useful to better understand the characteristics of binocular saccade coordination.
